# Young-onset frontotemporal dementia with FUS pathology

**DOI:** 10.1136/practneurol-2020-002730

**Published:** 2020-12-11

**Authors:** Matthew Gowell, Ian Baker, Olaf Ansorge, Masud Husain

**Affiliations:** 1 Medical Sciences Division, University of Oxford, Oxford, UK; 2 Russell Cairns Unit, Oxford University Hospitals NHS Foundation Trust, Oxford, Oxfordshire, UK; 3 Nuffield Department of Clinical Neurosciences, University of Oxford, Oxford, Oxfordshire, UK

**Keywords:** dementia, neuropsychology, histopathology, behavioural disorder

## Abstract

Frontotemporal dementia (FTD) is an uncommon cause of behavioural change in adults under the age of 50. A 44-year-old man presented with progressive neuropsychiatric disturbance characterised by social withdrawal, apathy, loss of empathy, motor stereotypies and hyperorality. Cognitive testing identified severe impairment, including executive dysfunction. MR scan of the brain showed bilateral symmetrical frontal atrophy. There was no relevant family history, and targeted genetic testing for FTD-associated variants in *MAPT*, *GRN* and *C9orf72* genes proved negative. He became more withdrawn with disinhibited behaviour; his condition progressively worsened and he died 6 years later. The pathological diagnosis was frontotemporal lobar degeneration with fused-in-sarcoma (FUS) pathology, a rare sporadic cause of FTD, accounting for only 5%–10% of cases, its characteristic features including very young onset, motor stereotypies and hyperorality.

## Clinical summary

A 44-year-old man was referred with a progressive history of changes in behaviour and personality, seemingly following a head-on collision with another car 6 months before. The other driver claimed that the patient had been driving erratically, straying to the wrong side of the road. There was no associated traumatic head injury. The patient’s partner described that he had suffered from low mood for at least 18 months before the event, after a close relative had died. He had been communicating less, being less sociable and noticeably less affectionate.

He had lost interest in performing activities and initiating social engagements. Instead, he spent most of his time watching television or browsing online, sometimes repeatedly watching the same programme. Six months before the car crash, he had lost his job because his employers were concerned that he was distracted and not completing work properly, frequently making uncharacteristic errors on routine tasks. At this time, his partner also observed that he ‘constantly had his hands down his trousers playing with himself’ and made repetitive movements seemingly without purpose, such as tapping his feet vigorously for long periods or placing inedible objects in his mouth. He also showed changes in dietary preference, with a new predilection to sweet foods.

There was no significant medical history. He had been prescribed citalopram since the day of the car crash for suspected depression. He smoked 20 cigarettes a day, drank alcohol only occasionally and took no recreational drugs. There was no family history of neuropsychiatric illness.

On examination, there was little spontaneous speech. He did not attempt to initiate conversation, answering questions with short, terse responses. He often gazed with a prolonged, fixed stare and exhibited motor stereotypies such as clapping his thighs or tapping his feet vigorously. There were no upper or lower motor neurone signs, no frontal release signs, and no parkinsonism or limb apraxia. His gait was normal. He scored only 55 out of 100 on the Addenbrooke’s Cognitive Examination-III, with impairments across domains but with particularly poor executive function (naming only two words beginning with P in a minute, and only 10 animals over a minute, leading to a very low verbal fluency score of 4 out of 14). Attention (score 12 out of 18), language (19 out of 26), memory (9 out of 26) and visuospatial (11 out of 16) abilities were also affected. Further neuropsychological testing identified a global reduction in general intellectual function, with severe executive function deficits as well as poor verbal working and episodic memory (box 1).

Box 1 **Neuropsychological test results**

- Verbal IQ=64.
- Performance IQ=72.
- Graded naming: 20/30 (within normal range).

**Executive function:**

- Letter fluency: first centile.
- Trail A: third centile; trail B: discontinued.
- Stroop: below the first centile.

Verbal working memory (digit span):

- 4 forwards; 3 backwards (very impaired).

Verbal episodic memory (immediate and delayed story recall):

- First and second centiles, respectively.

Visuoconstructive ability (copying complex figure):

- Accurate.

Visuospatial episodic memory (immediate and delayed recall of complex figure):

- 92nd and 81st centiles, respectively.

**Figure 1:**
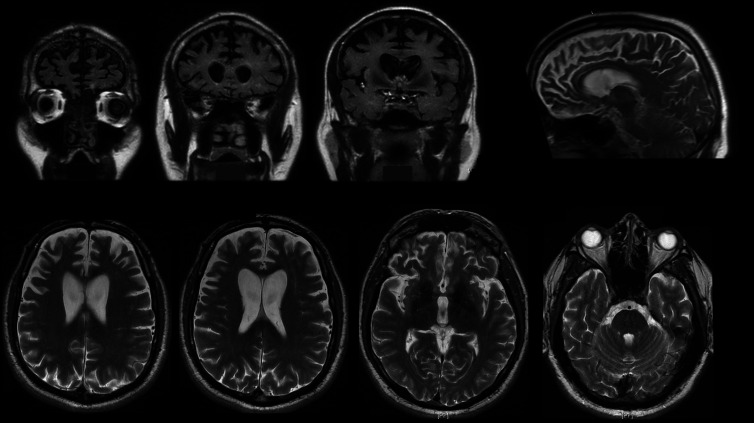
MRI. Coronal FLAIR (fluid-attenuated inversion recovery) sequences and sagittal T2-weighted imaging (top row) showed widespread lateral and medial frontal atrophy. Axial T2 images (bottom row) also showed symmetrical dilatation of the frontal horns of the lateral ventricles.

The differential diagnosis for such a progressive clinical presentation is quite extensive but can be reduced rapidly with some key investigations. First, in this case, an MR scan of the brain showed substantial bilateral, symmetrical frontal atrophy, with no evidence of white matter changes that would be typical of a vascular, vasculitic or inflammatory cause (figure 1). In addition, blood tests for C reactive protein; antinuclear, anticardiolipin and antiendomysial antibodies; and HIV and syphilis serology were all normal or negative. Cerebrospinal fluid (CSF) contained no white cells or abnormal cells and therefore no evidence of an active inflammatory, infective or malignant process; the CSF protein concentration was also normal. To investigate the (low) possibility of prion disease, real-time quaking-induced conversion was performed on the CSF and returned negative for prion protein. Finally, genetic testing for mutations in genes commonly associated with frontotemporal lobar degeneration (FTLD) (*MAPT*, *GRN*, *C9orf72*) was negative. The clinical findings and results of investigations satisfied the diagnostic criteria for probable behavioural variant frontotemporal dementia (bvFTD).^1^

The patient’s condition progressively worsened, with development of disinhibited, disruptive behaviour and eventual mutism. Trazodone, an atypical serotonergic agent that can reduce agitation, irritability, mood and eating disorder in patients with frontotemporal dementia (FTD),^2^ helped to improve his disinhibited and repetitive behaviours.

**Figure 2:**
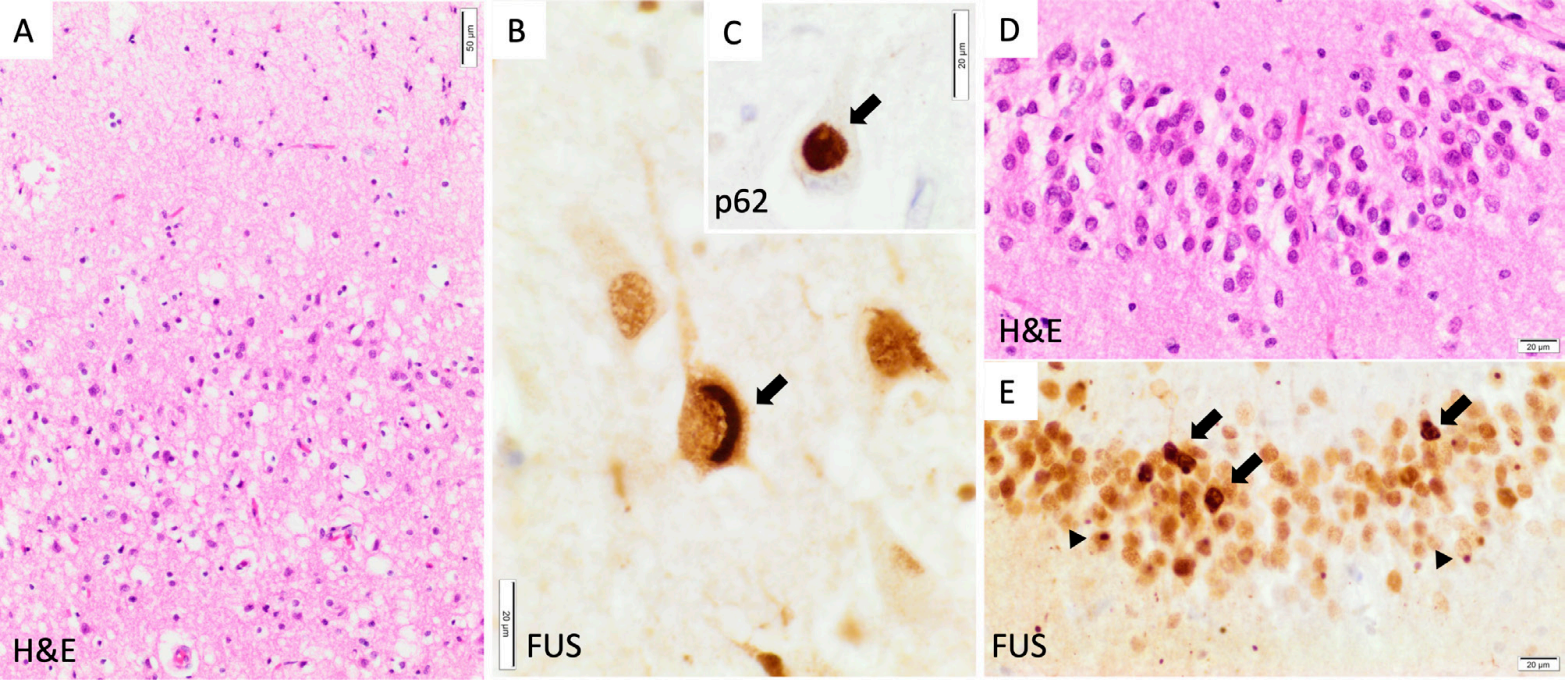
Neuropathology. Examination showed typical FTLD-FUS pathology. (A) Neuronal loss and spongiosis in the superficial layers of the prefrontal neocortex. (B) Crescent-shaped cytoplasmic FUS aggregate (arrow) in a pyramidal cell of CA1. (C) Globular cytoplasmic aggregate (arrow) stained for p62 in a pyramidal cell of CA1. (D) Mild depletion of the granule cells of the dentate gyrus of the hippocampus. (E) Numerous heterogeneous cytoplasmic (arrows) and nuclear as well as dot-like FUS inclusions (arrowheads) in the granule cells of the hippocampus. FUS: immunohistochemistry with FUS antibody (Sigma-Aldrich, HPA008784, 1:300); p62: immunohistochemistry with sequestosome-1 (p62) antibody (Abcam, ab91526, 1:1000). FTLD-FUS, frontotemporal lobar degeneration with fused-in-sarcoma pathology; FUS, fused-in-sarcoma.

The patient participated in the Brains for Dementia Research project, donating his tissue to the Oxford Brain Bank following his death, 6 years later. Macroscopic examination confirmed bilateral frontotemporal lobar atrophy without obvious asymmetry. Histopathology demonstrated neuronal loss and gliosis in the frontal and temporal cortex as well as hippocampal sclerosis. These areas contained neuronal and glial ubiquitin-positive inclusions which were also positive for fused-in-sarcoma (FUS) protein and the chaperone sequestosome-1 (p62) (figure 2). Immunostains for Aβ, alpha-synuclein, hyperphosphorylated (p)tau and pTDP-43 were negative. These features led to a neuropathological diagnosis of frontotemporal lobar degeneration associated with fused-in-sarcoma pathology (FTLD-FUS, atypical FTLD variant).

## Discussion

The pathological diagnosis of FTLD encompasses a heterogeneous group of neurodegenerative disorders predominantly affecting the frontal and anterior temporal lobes. While it manifests with an insidious onset and progression in all affected individuals, there are three clinical syndromes: semantic dementia, progressive non-fluent aphasia and bvFTD.^3^ Semantic dementia and progressive non-fluent aphasia both involve an initial decline in linguistic ability: patients with semantic dementia show deficits in object naming and word comprehension, whereas those with progressive non-fluent aphasia have impaired language production. bvFTD on the other hand is typified by early executive dysfunction and behavioural changes of disinhibition, apathy, loss of empathy or sympathy, motor stereotypies and hyperorality, and generally a fairly symmetrical pattern of FTLD.^4^ This patient had all of the typical clinical features of bvFTD, although he also developed a language component later.

The presence of behavioural features alone, however, is insufficient for a diagnosis of probable bvFTD. Some patients meet symptomatic criteria but have no evidence of FTLD on imaging and instead follow a benign course, known as ‘behavioural variant FTD phenocopy syndrome’.^5^ These individuals may have an underlying psychiatric disorder causing behavioural features that mimic bvFTD, sometimes making it difficult to distinguish between the two diagnoses. Indeed, apathy and emotional withdrawal were initially attributed to depression in our patient by his general practitioner. Repetitive and compulsive behaviours, however, would be unusual for major depression. Delusions and euphoria, also sometimes observed in bvFTD, may be wrongly diagnosed as late-onset schizophrenia.^6^

Given the marked cognitive deficits evident in this case, we also considered other causes of young-onset dementia. For example, an Alzheimer’s pathology can sometimes be distinguished clinically from FTLD by the presence of more prominent deficits in memory, visuospatial tasks and limb praxis, with relatively intact social behaviour and more generalised brain atrophy on imaging.^4^ However, it is now also recognised that there is a ‘frontal, dysexecutive’ variant of Alzheimer’s disease which can manifest similarly to bvFTD, with frontal atrophy on neuroimaging.^7^ Up to 40% of patients with FTLD may also develop features of motor neurone disease.^8^ However, this was not the case in our patient, who did not develop pyramidal or lower motor neurone signs over a 6-year follow-up.

In addition to syndromic classification, FTLD can also be divided into three histopathological subtypes, based on the nature and pattern of abnormal protein deposition within the brain: tau (FTLD-tau), TDP-43 (FTLD-TDP) or FUS (FTLD-FUS).^9^ In this patient, there was FUS (an RNA/DNA binding protein) pathology within the frontal and temporal cortex on autopsy. This is a rare finding, present in only 5%–10% of patients with FTLD.^10 11^ It is possible to correlate different pathologies with particular clinical presentations.^12^ Semantic dementia is almost always associated with TDP-43 proteinopathy, while progressive non-fluent dysphasia can be associated with tau or TDP-43. bvFTD, however, has been linked to all three histopathological subtypes, unless accompanied by motor neurone disease, in which case it is consistently linked with TDP-43 proteinopathy. Thus, it is often difficult to distinguish between pathologies on the basis of clinical features alone.^12^

Retrospective studies correlating pathology on autopsy with clinical features exhibited in life suggest there might be some features that point to a diagnosis of FTLD-FUS. Typically, bvFTD presents in the sixth decade of life, but in patients with FUS pathology onset is considerably younger, often in the fourth decade.^13^ Other pathologies are also possible in younger patients though.^14^ Hyperorality and dietary change in bvFTD are associated with both FUS and tau pathologies, but typically not TDP-43.^15^

There was no family history of neuropsychiatric illness in our case. Of FTD cases 60% present sporadically (although it is important to bear in mind the possibility of gene mutations in individuals with cryptic family histories).^16^ The remainder show an autosomal dominant pattern of inheritance, of which 60% are caused by mutations in *MAPT*, *C9orf72* and *GRN*.^17^ In recent years, mutations in particular genes, including *MAPT* and *C9orf72*, have been correlated with tau and TDP-43 proteinopathies, respectively. However, no mutations have yet been linked with the FTLD-FUS histological subtype (which causes behavioural change without motor neurone signs), including in the *FUS* gene itself (*FUS* was not sequenced in our case).^18^ Instead, *FUS* mutations, which are always accompanied by FUS protein inclusions, are linked to a specific form of amyotrophic lateral sclerosis (ALS-FUS).^19^ However, despite shared FUS pathology, cases of ALS-FUS appear to have a different underlying pathogenic mechanism from sporadic FTLD-FUS.

This case is an example of neuropsychiatric and cognitive disturbance in a young patient who was diagnosed with bvFTD with underlying rare FTLD-FUS pathology. Clinical features and investigations pointed to a diagnosis of probable bvFTD. Young onset accompanied by hyperorality and dietary changes can occur with several underlying pathologies, but recent work suggests these are often present in patients with FTLD-FUS pathology.

**Key points**

- Progressive behavioural change is most likely to be attributed to an underlying psychiatric cause, but if associated with significant cognitive impairment should raise the possibility of frontotemporal dementia (FTD).
- Apathy, loss of sympathy or empathy, perseverative stereotyped or compulsive behaviour, hyperorality, and dietary change are important clues and key features of behavioural variant FTD.
- Young-onset (<50 years of age) behavioural variant FTD raises the possibility of fused-in-sarcoma (FUS) pathology.
- If there are no associated signs of motor neurone disease, this is highly unlikely to be an inherited mutation in the *FUS* gene.

**Further reading**

- Warren JD, Rohrer JD, Rossor MN. Clinical review. Frontotemporal dementia. *BMJ* 2013;347:f4827.
- Neumann M, Mackenzie IRA. Review: Neuropathology of non-tau frontotemporal lobar degeneration. *Neuropathol Appl Neurobiol* 2019;45:19–40.
- Mann DMA, Snowden JS. Frontotemporal lobar degeneration: pathogenesis, pathology and pathways to phenotype. *Brain Pathol* 2017;27:723–36.
